# Effects of zero reference position on bladder pressure measurements: an observational study

**DOI:** 10.1186/2110-5820-2-S1-S13

**Published:** 2012-07-05

**Authors:** Caridad De Dios Soler Morej&#243n, Tomás Ariel Lombardo, Teddy Osmin Tamargo Barbeito, Barquín García Sandra

**Affiliations:** 1 Intensive Care Unit, Hermanos Ameijeiras Hospital, San Lázaro and Belascoaín, La Habana, 10300, Cuba; 2 Department of Surgery, General Calixto García Teaching Hospital, Vedado, La Habana, 10400, Cuba; 3 Bioestatistical Medicine, Department of Research and Development, Hermanos Ameijeiras Hospital, San Lázaro and Belascoaín, La Habana, 10300, Cuba; 4 Intensive Care Unit, General Calixto García Teaching Hospital, Vedado, La Habana, 10400, Cuba

**Keywords:** intra-abdominal pressure, measurement, bladder pressure, zero reference, midaxillary line, symphysis pubis, laparotomy.

## Abstract

**Background:**

Although the World Society for Abdominal Compartment Syndrome in its guidelines recommends midaxillary line (MAL) as zero reference level in intra-abdominal pressure (IAP) measurements in aiming at standardizing the technique, evidence supporting this suggestion is scarce. The aim of this study is to study if the zero reference position influences bladder pressure measurements as estimate for IAP.

**Methods:**

The IAP of 100 surgical patients was measured during the first 24 h of admission to the surgical intensive care unit of General Calixto Garcia Hospital in Havana (Cuba) following laparotomy. The period was January 2009 to January 2010. The IAP was measured twice with a six-hour interval using the transurethral technique with a priming volume of 25 ml. IAP was first measured with the zero reference level placed at MAL (IAP_MAL)_, followed by a second measurement at the level of the symphysis pubis (SP) after 3 minutes (IAP_SP_). Correlations were made between IAP and body mass index (BMI), type of surgery, gender, and age.

**Results:**

Mean IAP_MAL _was 8.5 ± 2.8 mmHg vs. IAP_SP _6.5 ± 2.8 mmHg (*p *< 0.0001). The bias between measurements was 2.0 ± 1.5, 95% confidence interval of 1.4 to 3.0, upper limit of 4.9, lower limit of -0.9, and a percentage error of 35.1%. IAP_MAL _was consistently higher than IAP_SP _regardless of the type of surgery. The BMI correlated with IAP values regardless of the zero reference level (*R*^2 ^= 0.4 and 0.3 with IAP_MAL _and IAP_SP _respectively, *p *< 0.0001).

**Conclusions:**

The zero reference level has an important impact on IAP measurement in surgical patients after laparotomy and can potentially lead to over or underestimation. Further anthropometric studies are needed with regard to the relative MAL and SP zero reference position in relation to the theoretical ideal reference level at midpoint of the abdomen. Until better evidence is available, MAL remains the recommended zero reference position due to its best anatomical localization at iliac crest.

## Introduction

The bladder is the gold standard for noninvasive indirect intra-abdominal pressure (IAP) measurement [1-8]. In 1984, Kron et al. described originally the technique as an open system for single IAP measurement at the bedside using the symphysis pubis (SP) as a zero reference level and with IAP expressed in centimeters of water [9]. Over the last ten years, midaxillary line (MAL) has been used as the zero reference level when measuring IAP.

Since Kron's report, new closed techniques have been suggested which minimize the risk of urinary tract infection and workload while improving accuracy and reproducibility [1,4,10-12].

Reproducibility of IAP measurements can be affected by many factors. Among the most important is the positioning of the pressure transducer with regard to the reference level. It is well known that this may under or overestimate IAP [4,13].

Measuring IAP with accuracy and reproducibility is of utmost importance since treatment may depend on it. In this paper, IAP values were measured in post-laparotomy patients at MAL reference level and compared to those obtained at SP.

### Patients and methods

This is an observational study comparing two different reference levels for measuring IAP in 100 surgical patients. The study was conducted in the surgical intensive care unit at General Calixto García Hospital between January 2009 and January 2010. This facility is a level 3 university hospital. The patients were included if they were admitted within 24 h after laparotomy. Criteria used to exclude patients were: bladder surgery or any contraindication to measure IAP via the bladder, pregnancy, 'open abdomen' after laparotomy, neurological disorders affecting the bladder, surgical intraoperative complications (bleeding and visceral injuries), re-laparotomy, mechanical ventilation, and patients under 16 years old.

**Figure 1:**
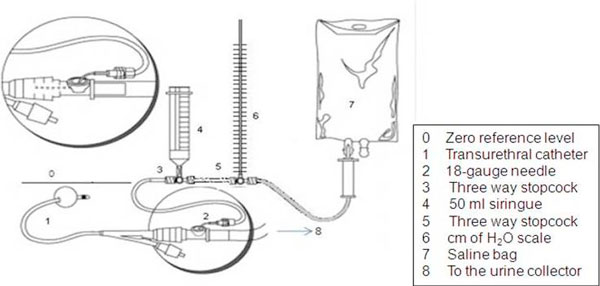
**IAP measurement technique**. A centimeters of water scale is inserted instead of a transducer, an adaptation of Cheatham and Safsack's technique [11] (reprinted with permission from the author).

IAP was measured in each patient according to Cheatham and Safsack's technique [11] and World Society on Abdominal Compartment Syndrome (WSACS) guidelines and recommendations for research [3,5,6]. Instead of using a transducer, a scale in centimeters of water or cmH_2_O was added to the urinary drainage system (Figure 1). Two measurements at end expiration with a six-hour interval were made at each zero reference level during the first 24 h by the same nurse in order to avoid interobserver variability. The intrabladder saline volume was 25 ml. In the supine position, the scale was zeroed at the MAL using the superior iliac crest as reference point. Another measurement was taken, this time using SP as reference level, 3 min after the first one in order to allow correct calibration of the system and bladder detrussor muscle relaxation. During the measurement no sedatives were used, but analgesic drugs were provided when necessary. Each IAP value was obtained with (cmH_2_O) and recalculated in millimeters of Mercury or mmHg, using the conversion factor (1 cmH_2_O = 0.74 mmHg). The two IAP values obtained at each reference level with the 6-hour interval were averaged and the results were entered in a database. The total number of measurements was 400.

Intra-abdominal hypertension (IAH) was defined when mean intra-abdominal pressure at midaxillary line (IAP_MAL_) was ≥12 mmHg. According to their body mass index (BMI), the patients were stratified in four groups: less than 20 kg/m^2^, 20 to 25 kg/m^2^, 26 to 30 kg/m^2 ^and more than 30 kg/m^2^.

### Data management and statistical analysis

The Statistical Package for Social Sciences (SPSS for Windows version 16.0 software SPSS Inc., Chicago, IL, USA) was used in order to organize, validate, and analyze the collected data. The indicators of central tendency and dispersion are the following: medians, means, standard deviations (SD), interquartile range (IQR) and 95% confidence intervals (CI) were estimated for quantitative variables, while frequencies and percentages were used for qualitative variables. A two-sample paired *t *test was used to evaluate differences of means in two samples and normality assumption, and Wilcoxon signed-rank test was used to investigate the differences of means in two paired samples and non normality assumption. The Mann-Whitney *U *test was used to evaluate the differences of means in two independent samples and non normality assumption. The one-way analysis of variance was used to compare more than two means. Chi-squared test with Yates's correction for continuity or Fisher's exact test was used wherever appropriate in order to identify the differences between categorical variables. In addition, IAP values obtained in the different levels were compared using Bland and Altman analysis, Pearson, and intra-class correlation coefficient (ICC). Pearson correlation test was also applied to find out any association between BMI and IAP.

A confounder adjustment with logistic regression model was performed to analyze the influence of age, BMI, IAP_MAL_, gender and type of surgery on the probability of death in intensive care unit (ICU). A *p *value of < 0.05 was considered to be significant for all the statistical tests. Tables and figures were constructed in order to present the most relevant findings. The primary endpoint in the study was the difference between the measured IAP values at MAL and SP references levels.

The study protocol was approved by the local ethics committee and institutional review board (53/2008). Informed consent had been obtained from the patient or next of kin before their inclusion in this study. The IAP measurement did not interfere with other procedures, according to the recommendations of the Council for International Organizations of Medical Sciences [14] and of the Helsinski Declaration [15].

## Results

One hundred (100) post-abdominal surgery patients were included in this study, 52% of whom were females. The age was 53.5 ± 16.1 years. Fifty-five (55) patients had undergone emergency operations of which bowel obstruction had been the most common pathology (28 emergency patients, 51%); 45 had had elective surgery and among them 24 (53%) had received bariatric surgery. According to the stated definition, nine patients had IAH. There were no patients with abdominal compartment syndrome (ACS). The BMI was higher in patients with IAH (40.4 ± 8.7 mmHg vs. 25.9 ± 9.2 mmHg) (*p *< 0.0001). Acute Physiology and Chronic Health Evaluation (APACHE) II and Sequential organ failure assessment (SOFA) scores are also showed. The ICU mortality was 15% and no IAH patients died during their length of stay in this unit (Table 1).

**Table 1:** Characteristics of the patients (*n *= 100)

	Total (*n *= 100)	IAH (*n *= 9)	Non-IAH (*n *= 91)	*p *value
Female gender	52 (52.0%)	8 (88.9%)	44 (48.4%)	0.032^a^
Age (years)	53.5 ± 16.1	38.1 ± 2.5	55.0 ± 16.0	0.001^b^
BMI (kg/m^2^)	27.2 ± 10.0	40.4 ± 8.7	25.9 ± 9.2	< 0.0001^b^
Emergency surgery	55 (55.0%)	0 (0%)	55 (60.4%)	< 0.0001^a^
Bariatric surgery	24 (24.0%)	9 (100%)	15 (16.5%)	< 0.0001^a^
APACHE II	11.2 ± 1.7	12.1 ± 2	10.3 ± 1.5	0.183^b^
SOFA	4.5 ± 2.8	5.1 ± 3.6	4.0 ± 2.0	0.006^b^
ICU mortality	15 (15%)	0 (0.0%)	15 (16.5%)	0.348^a^
IAP_MAL _(mmHg)	8.5 ± 2.8	12.9 ± 0.6	8.1 ± 2.5	< 0.0001^b^

^a^Fisher's exact test; ^b^Mann-Whitney *U *test. APACHE, Acute Physiology And Chronic Health Evaluation II; BMI, body mass index; IAH, intra-abdominal hypertension; IAP_MAL_, intra-abdominal pressure at midaxillary line; SOFA, Sequential organ failure assessment.

**Figure 2:**
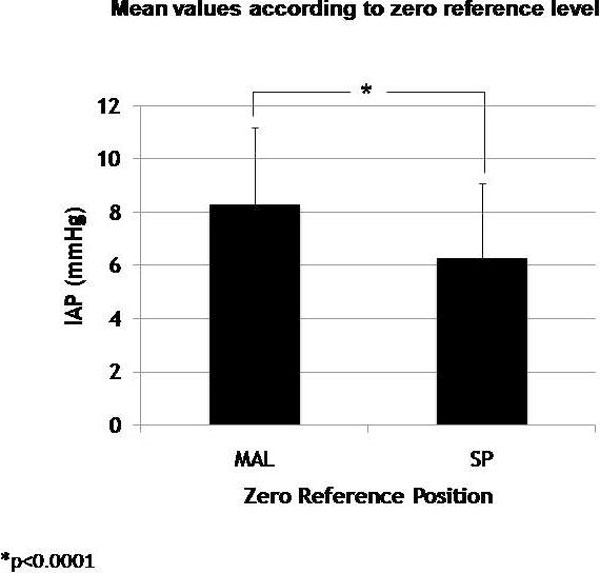
**Mean IAP values (mmHg) according to the reference level of measurement (MAL or SP)**. Asterisk indicates paired student *t *test. IAP, intra-abdominal pressure; MAL, midaxillary line; SP, symphysis pubis.

The mean IAP_MAL _values were 8.5+/-2.8 mmHg (95% CI 7.9 to 9.0). At SP, mean IAP was 6.5 ± 2.8 (95% CI 5.9 to 7.0) (Figure 2). The paired student *t *test revealed a significant difference between them (*p *< 0.0001).

**Figure 3:**
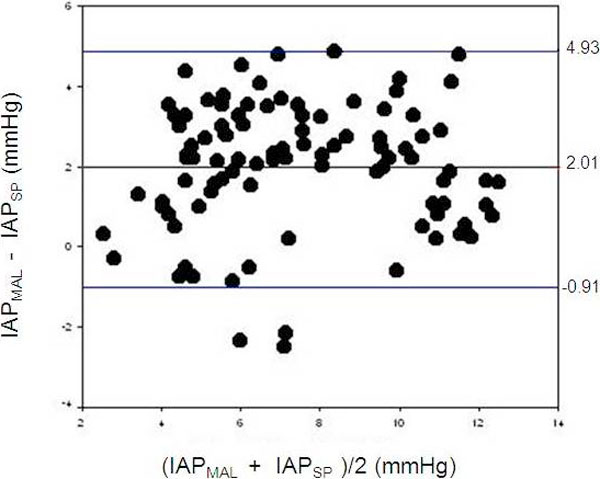
**Bland and Altman analysis**. According to Bland and Altman analysis, the calculated bias was 2.01; precision at 1.49; two SD of 2, 98; limits of agreements -0.91 to 4.93; and error of 35.1%. This percentage of error is too high (percentage of error recommended is 25%). IAP, intra-abdominal pressure; MAL, midaxillary line; SP, symphysis pubis.

According to Bland and Altman analysis, the bias between IAP measurements was 2.0 with a precision of 1.5 and a percentage error of 35.1% (upper limit and lower limits of agreement of 4.9 and minus 0.9, 95% CI 1.4 to 3.0). Almost all the values are included in the range of concordance (Figure 3). In the correlation according to Pearson, comparison was good (*R*^2 ^= 0.64, *p *< 0.0001) and ICC was 0.84, *p *= 0.001 (95% CI 0.7 to 0.8).

**Figure 4:**
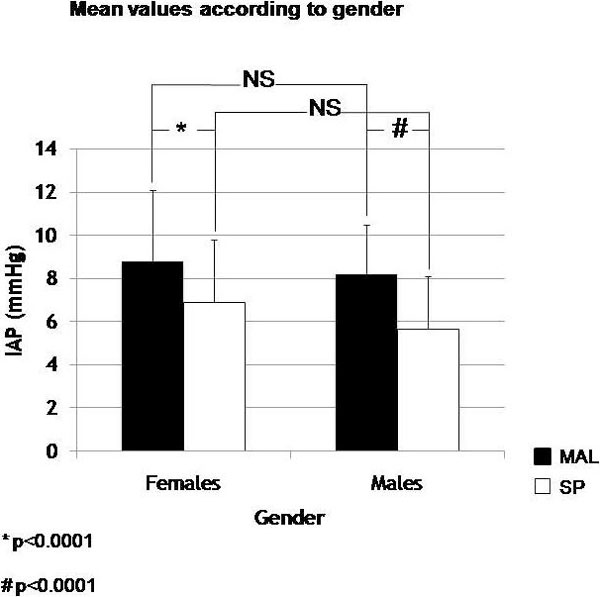
**IAP values according to gender in both reference levels**. Asterisk indicates two-sample paired *t *test; Number sign indicates two samples paired *t *test. IAP, intra-abdominal pressure; MAL, midaxillary line; NS, non-significant; SP, symphysis pubis.

There was a significant difference in the mean IAP at each reference level within each gender (*p *< 0.0001, 95% CI 1.4 to 2.2 females, and 95% CI 1.8 to 2.6 males), but there was no difference when using the same reference point (Figure 4).

BMI was positively correlated with IAP in both reference levels (*R*^2 ^= 0.4 and 0.3, *p *< 0.0001). According to the given definition, only three BMI categories were identified in the study population as there were no patients under 20 kg/m^2 ^(Table 2). IAP values increased according to BMI for the two reference levels. One-way analysis of variance showed significant differences between groups (*p *< 0.0001).

**Table 2:** BMI categories and mean IAP values

BMI (kg/m^2^)	IAP_MAL _(mmHg)	IAP_SP _(mmHg)	*p *value
20-25 (*n *= 68)	7.4 ± 2.2	5.4 ± 2.1	< 0.0001^a^
26-30 (n = 8)	7.4 ± 2.3	5.6 ± 2.8	0.012^b^
> 30 (*n *= 24)	12.1 ± 0.8	10.0 ± 1.3	< 0.0001^b^
*p *value^c ^	< 0.0001	< 0.0001	

^a^Two-sample paired *t *test; ^b^Wilcoxon signed-rank test; ^c^one-way analysis of variance. BMI, body mass index; IAP, intra-abdominal pressure; MAL, midaxillary line; SP, symphysis pubis.

In addition, mean IAP values revealed a significant difference according to the zero reference position (Table 2). BMI was positively correlated to IAP regardless of reference level.

The IAP values in elective surgery were higher than those in emergency surgery (IAP_MAL _7.4 ± 1.9 vs. 9.8 ± 3.1 and intra-abdominal pressure at symphysis pubis (IAP_SP_) 5.4 ± 2 vs. 7.8 ± 2.9). The highest IAP values were measured in the bariatric group. This group had also the highest BMI values. Regardless of the type of surgery, IAP_MAL _was always significantly higher than IAP_SP _(Table 3).

**Table 3:** BMI and IAP values in regard to the type of surgery

Type of surgery		BMI (kg/m^2^)	IAP (mmHg)			
			MAL	SP	*p *value	CI (95%)
Emergency (*n *= 55)		22.1 ± 2.1	7.4 ± 1.9	5.4 ± 2.0	< 0.0001^b^	1.5-2.4
Elective	Bariatric (*n *= 24)	42.4 ± 10.0	12.1 ± 0.8	10.0 ± 1.3	< 0.0001^b^	1.5-2.6
	Non-bariatric (*n *= 21)	23.1 ± 2.5	7.1 ± 2.7	5.4 ± 2.4	0.002^b^	0.7-2.6
	Total (*n *= 45)	30.5 ± 18.1^a^	9.8 ± 3.1	7.8 ± 2.9	< 0.0001^b^	1.6-2.4

^a^Median ± IQR; ^b^Two-sample paired *t *test;^c^Wilcoxon signed-rank test; BMI, body mass index; IAP, intra-abdominal pressure; MAL, midaxillary line; SP, symphysis pubis.

Fifteen patients in the ICU died after complications. Complications arose in 11 patients after emergency surgery and in 4 patients following surgery for malignancy. The most frequent complication was pneumonia (*n *= 7) followed by septic shock (*n *= 5). One patient died from multiple organ failure; another developed adult respiratory distress syndrome, and one patient died of hypovolemic shock. The IAP_MAL _did not differ significantly between survivors and non-survivors (*p *= 0.5) and neither did IAP_SP _(*p *= 0.74) (Table 4), so IAP_MAL _was higher than IAP_SP _regardless of the outcome. No bariatric patients died during their length of stay in ICU, and the majority of the decedents (11) were emergency patients (Table 4).

**Table 4:** Characteristics of the patients according to ICU mortalities

	Total(*n *= 100)	ICU mortalities		
		Survivors (*n *= 85)	Non-survivors(*n *= 15)	*p *value
Female gender	52 (52.0%)	46 (54.1%)	6 (40.0%)	0.466^a^
Age (years)	53.5 ± 16.1	51.5 ± 15.7	64.7 ± 13.8	0.003^b^
BMI (kg/m^2^)	27.2 ± 10.0	27.9 ± 10.7	23.3 ± 2.8	0.508^b^
Emergency surgery	55 (55.0%)	44 (51.8%)	11 (73.3%)	0.205^a^
Bariatric surgery	24 (24.0%)	24 (28.2%)	0 (0.0%)	0.019^c^
APACHE II	11.2 ± 1.7	9.3 ± 1.5	13.1 ± 2.0	< 0.0001^b^
SOFA	4.5 ± 2.8	3.9 ± 2.2	6.4 ± 3.4	0.021^b^
IAP_MAL _(mmHg)	8.5 ± 2.8	8.1 ± 2.4	8.6 ± 2.8	0.505^b^
IAP_SP _(mmHg)	6.5 ± 2.8	6.3 ± 3.0	6.6 ± 2.7	0.743^b^

^a^Chi square test with Yates's correction for continuity; ^b^Mann-Whitney *U *test; ^c^Fisher's exact test. APACHE, Acute Physiology and Chronic Health Evaluation II; BMI, body mass index; IAH, intra-abdominal hypertension; IAP_MAL_, intra-abdominal pressure at midaxillary line; IAP_SP_, intra-abdominal pressure at symphysis pubis; SOFA, Sequential organ failure assessment.

A multivariate analysis showed that the only independent factors associated with mortality were age and BMI (Table 5). Concerning IAP, there was a non-significant tendency toward higher values in non-survivors.

**Table 5:** Logistic regression analysis of risk factor for ICU mortalities

Independent variables	Odds ratio	95% CI	*p *value
Age	1.053	1.005-1.103	0.030
BMI	1.321	1.010-1.728	0.042
IAP_MAL_	1.321	0.965-1.810	0.083
Gender^a^	1.695	0.461-6.236	0.427
Type of surgery	(Dummy)^b^		
Bariatric	0.000	0.000-0.000	0.996
Non-bariatric	0.746	0.176-3.164	0.691

Reference categories: ^a^female; ^b^emergency; BMI, body mass index; CI, confidence interval; IAP_MAL_, intra-abdominal pressure at midaxillary line.

## Discussion

The prevalence of IAH in this cohort population was low in comparison with previous studies [16,17], and no ACS was identified. In the present study, the patient population was less severely ill and included only post-laparotomy patients who did not have surgical intraoperative complications on admission or were not mechanically ventilated, with low APACHE II and SOFA scores.

IAP values in the post-laparotomy patients were significantly higher when measured at MAL compared to SP. This finding is in accordance to previously published studies. In 2008, the WSACS clinical trials group reported the results of a multicenter prospective trial aimed to investigate the effect of three different reference transducer positions on IAP measurement. They found that IAP_MAL _was higher than IAP phlebostatic and IAP_SP_, and the differences were clinically significant (*p <*0.001) [13].

According to the ICC, the mean IAP_MAL _compared to IAP_SP _were not significantly different, implying that the variation of the measurements were dependent on the patients' characteristics [18,19]. Although IAP measured at MAL or at SP can be similar in its efficacy, according to Bland and Altman analysis, bias and percentage error were too large to consider both reference points equivalents, so IAP should always be measured at the same reference level to avoid additional sources of bias during the measurement.

Unless more evidence becomes available, it would be recommended to use the MAL at iliac crest as zero reference level, as stated previously [3-6,13,20,21], because it offers a better anatomical reference. The SP reference level is generally placed according to imprecise anatomical details due to differences in patient's body habitus.

IAP was significantly higher as BMI increased in both reference levels regardless of the type of surgery. This relation between BMI and IAP has been reported in previous studies in non-critically ill patients by Sánchez et al. [22] and Noblett et al. [23], and in critically ill patients by Soler [7]. On the other hand, BMI was identified by Malbrain et al. as an independent factor for IAH in a multicenter study. De Keulenaer et al. propose that normal IAP values in obese patients should be considered between 7 and 14 mmHg and hypothesize that higher pressure in the obese patients are due to the direct effect of the intra-abdominal adipose tissue itself on the measurement [12]. In this study the IAP values in the obese patients were similar to those suggested by the same author.

The age and the BMI were the only independent factors that could be identified in this study in relation to mortality as shown in the multivariate analysis. The post-laparotomy patients in this cohort didn't have intraoperative complications at admission and their SOFA and APACHE II scores were relatively low. The IAP was not significantly related to mortality, though there was a trend toward higher values in non-survivors. As reported before in two multicenter studies, the mean IAP on admission is not considered as an independent risk for mortality [16,17].

According to Malbrain, some factors may influence the diagnostic accuracy of the IAP measurement and provoke inter or intraobserver variability [1,4] such as changes in patient's position without repositioning the pressure transducer level, prone position, distortions or artifacts in IAP wave form (over-or under-damping), and air bubbles in the connections. Among them, the effects of the body position on IAP measurements are relevant [12,24]. Other factors are the intrinsic bladder compliance and the amount of instilled fluid in the bladder [1,4].

What is considered as the best reference level is also a controversial subject. In accordance with the opinion of some authors, the ideal zero position should be placed at the tip of the Foley catheter [13], but this can introduce a measurement bias. On the other hand, a more logical approach could be to set it at midpoint of the abdomen as suggested previously [21]. Thus, to state the best zero reference position may depend on anthropometric factors such as BMI, sagittal abdominal diameter, etc. Those factors cannot be easily modified at the bedside of a critically ill patient.

The factors influencing the accuracy and reproducibility of IAP measurements were discussed during the World Congress on Abdominal Compartment Syndrome in December 2004 in Australia. Later, a consensus on definitions and recommendations was published [5,6]. Based on these guidelines, the transducer position should be placed at the midaxillary line at the level of the iliac crest, as this anatomical reference could be the best alternative at the bedside. Compared to SP, this reference is less variable and easy to identify in any critically ill patient, obese or not and probably reflects the midpoint of abdomen.

Although this study can contribute to the effort of ensuring an accurate and reproducible measurement from patient to patient, it has some limitations. All values obtained in centimeters of water were recalculated to be expressed in mmHg. No body anthropomorphic data were collected to define the theoretical absolute zero point of the abdomen. Finally, the results obtained could not be extrapolated to other patient populations because the study was performed in a single hospital population of patients following laparotomy.

## Conclusions

This study, performed in 100 post-laparotomy patients, is the second next to the WSACS' multicenter trial [13] on this topic and confirms that the zero reference position influenced IAP values; therefore, IAP should always be measured at the same reference level. Further anthropometric studies are needed with regard to the relative MAL and SP zero reference position in relation to the theoretical ideal reference level at midpoint of abdomen. Until better evidence is available, MAL should be recommended as the zero reference position due to its best anatomical location at the iliac crest.

## Consent

Written informed consent was obtained from the patient or next of kin before their inclusion in this study. A copy of the written consent is available for review by the Editor -in-chief of this journal

## Abbreviations

ACS: abdominal compartment syndrome; APACHE: Acute Physiology and Chronic Health Evaluation; BMI: body mass index; CI: confidence interval; IAH: intra-abdominal hypertension; IAP: intra-abdominal pressure; IAP_MAL_: intra-abdominal pressure measured at midaxillary line; IAP_SP_: intra-abdominal pressure measured at symphysis pubis; ICC: intra-class coefficient; ICU: intensive care unit; IQR: interquartile range; MAL: midaxillary line; SD: standard deviation; SOFA: Sequential organ failure assessment; SP: symphysis pubis; WSACS: World Society on Abdominal Compartment Syndrome.

## Competing interests

The authors declare that they have no competing interests.

## Authors' contributions

CS was involved in the design, analysis and interpretation of data, and drafted the manuscript. AL collaborated in the design, interpretation of data, and critical revision of the manuscript. SB collected all data (IAP measurements and its recording). TT revised meticulously the data base and made a new statistical analysis. All authors read and approved the final manuscript.
